# Photoprotection for Skin Cancer: What’s New

**DOI:** 10.3390/cancers18040634

**Published:** 2026-02-15

**Authors:** Yolanda Gilaberte, Andrés Ederra-Galé, Juan J. García-Alfonso, Tamara Gracia-Cazaña

**Affiliations:** 1 Department of Dermatology, Miguel Servet University Hospital, 50009 Zaragoza, Spain; ygilaberte@gmail.com (Y.G.); ederratauste99@gmail.com (A.E.-G.); juanjozarandieta@gmail.com (J.J.G.-A.); 2 University of Zaragoza, 50009 Zaragoza, Spain

**Keywords:** photoprotection, sunscreens, oral photoprotection, antioxidants, keratinocyte skin cancer, non-melanoma skin cancer, melanoma, cutaneous squamous cell carcinoma, basal cell carcinoma

## Abstract

*Background*: Skin cancer is the most common malignancy worldwide. Although photoprotection is the cornerstone of skin cancer prevention, evidence regarding the role of other radiations different from the ultraviolet radiation and the efficacy of sunscreens, oral supplements, DNA-repair enzymes, and antioxidants continues to evolve. *Objectives*: To review the current evidence on photoprotective strategies and assess their role in preventing actinic keratoses (AK), keratinocyte carcinomas and melanoma. *Methods*: A narrative review of the literature was conducted using PubMed (2010–2025), including studies in English and Spanish. Search terms comprised “photoprotection,” “sunscreen,” “oral photoprotection,” “skin cancer,” “melanoma,” “keratinocyte cancer,” “UV radiation,” “non-melanoma skin cancer” and related concepts. Articles were selected for clinical relevance. *Results*: Daily sunscreen use significantly reduces AK and cutaneous squamous cell carcinoma in high-risk individuals, although evidence for basal cell carcinoma and melanoma prevention remains heterogeneous. Balanced broad-spectrum protection, including UVA and visible light filtering, seems to be important, whereas high-energy visible light needs further investigation. DNA-repair enzymes have shown reductions in cyclobutane pyrimidine dimers and clinical improvement of AK. Antioxidants such as *Polypodium leucotomos* extract, topical and oral, exhibit preventive effects on actinic damage and carcinogenesis. Topical and especially oral nicotinamide demonstrate chemopreventive potential in immunocompetent patients. Vulnerable populations—including transplant recipients, XP patients, individuals with albinism, and outdoor workers—require tailored photoprotection strategies with demonstrated benefit. *Conclusions*: Photoprotection extends far beyond UV filters, encompassing biological ingredients, antioxidants, oral supplements, and broad-spectrum strategies that target the full exposome. Comprehensive, behaviour-based photoprotection programmes are essential for high-risk groups.

## Simple Summary

Skin cancer is the most common cancer worldwide, and excessive exposure to sunlight is its main preventable cause. This review summarizes current evidence on sunscreens, oral supplements, antioxidants, and DNA-repair enzymes for preventing actinic keratoses and skin cancer. Regular daily sunscreen use reduces actinic keratoses and squamous cell carcinoma, particularly in individuals at high risk, while additional strategies may provide further benefit. Emerging evidence also suggests that visible light and other environmental factors contribute to skin damage and may play a role in skin carcinogenesis. The findings discussed here highlight the importance of comprehensive and individualized photoprotection strategies to improve skin cancer prevention and guide future research and clinical practice.

## 1. Introduction

Skin cancer is the most frequently diagnosed cancer worldwide [1]. The most frequently diagnosed include keratinocyte cancers (KC), basal cell carcinoma (BCC) and squamous cell carcinoma (SCC), and the most aggressive one, melanoma. The Global Burden of Disease study showed that the number of skin cancer cases increases every year, mostly due to the aging of the population and as a result of sun exposure especially in fair-skinned people [2]. For that reason, melanoma incidence is very high in countries with very high solar ultraviolet (UV) radiation levels, such as Australia and New Zealand. However, its incidence is also high in countries with high latitude, like northern Europe. In this case, it is thought that intense holiday sun exposure or the use of sunbeds can have a role [3]. Recent publications indicate that while KC incidence seems to have stabilized across most of Europe, cases are increasing among younger individuals, and SCC mortality continues to rise in older men [4].

To prevent skin cancer, photoprotection is recommended. This involves avoiding midday sun exposure; wearing protective clothing, hats, and sunglasses; seeking shade; and complementing these measures with the use of sunscreen.

## 2. Material and Methods

We conducted a narrative review of the literature. We performed literature searches with PubMed from January 2010 to December 2025 using the keywords “photoprotection”, “sunscreen”, “oral photoprotection”, “skin cancer”, “cutaneous cancer”, “melanoma”, “keratinocitic cancer”, “non-melanoma skin cancer”, “ultraviolet radiation”, “sunlight” and “solar radiation”. The search was limited to English and Spanish. Articles were selected depending on their clinical relevance.

## 3. Results

### 3.1. Efficacy of Sunscreens and Other Phoprotective Measures to Prevent Skin Cancer

In this section, we refer specifically to conventional topical sunscreens whose primary mechanism of action relies on UV filters (organic and/or inorganic) that absorb, reflect, or scatter UV radiation. The evidence discussed below evaluates the protective effect of these filter-based formulations on skin cancer prevention, independently of the presence of additional non-filtering photoprotective ingredients.

Sunscreen is widely assumed to be effective in preventing skin cancer; however, the scientific evidence is supportive but not conclusive. In this context, Waldman et al. [1] reviewed the only four prospective studies that assess this effectiveness [5,6,7,8]. Notably, the Nambour Skin Cancer Prevention Trial was the most significant population-based longitudinal clinical trial to date, as it enrolled 1621 participants who were randomly assigned to either discretionary use or daily application of sunscreen with an SPF > 15 over a period of 4.5 years [8]. Subsequently, follow-up assessments were conducted at 8 [9] and 10 years [10] after the initial study period. Daily sunscreen use was associated with a 39% reduction in cutaneous squamous cell carcinoma (cSCC) incidence after 4.5 years (OR 0.61; 95% CI, 0.46–0.81) and a 38% reduction after 8 years (RR 0.62; 95% CI, 0.38–0.99). In contrast, no significant differences were observed in the number of BCC cases, although a 25% decrease in the rate of BCC development was noted after 8 years [8,9]. At the 10-year follow-up, a borderline significant 50% reduction in melanoma incidence was observed among daily sunscreen users (HR 0.50; 95% CI, 0.24–1.02; *p* = 0.051), with a 73% reduction in invasive melanomas across all body sites (HR 0.27; 95% CI, 0.08–0.97; *p* = 0.045) and a lower mean Breslow thickness compared with discretionary users (0.53 vs. 1.2 mm) [10].

Nevertheless, a 2025 review examining the association between sunscreen use and cSCC reported inconsistent results both within and across 10 studies published between 1995 and 2022, thus preventing the authors from establishing any conclusive or generalizable conclusion [11]. Among the main limitations were differences in study design, geographic location, errors in sunscreen application and the inclusion of non-users of sunscreen (presumed to have a lower baseline risk of cSCC) as the control group. In the case of BCC, the lack of significance of the studies may be attributed to its slow progression, whereas for melanoma, it is likely related to its lower incidence compared to KC, resulting in limited statistical power rather than a true absence of efficacy. Additionally, participants in the control group were allowed to use sunscreen at their discretion.

Regarding the preventive effect of using sunscreen with higher versus lower SPF values, in the NOWAC cohort—which included more than 148,000 Norwegian women, aged 31 to 70 years, followed for an average of 14.3 years—a total of 653 cSCC were diagnosed, predominantly on the head and among older participants [12]. Most women (85%) reported regular sunscreen use, particularly younger women with fair skin and hair, a history of sunburns, and greater cumulative sun exposure. However, the effect of using sunscreen with SPF ≥ 15 compared to SPF < 15 on the risk of developing cSCC was essentially null, both in the overall analysis (HR 1.02; 95% CI, 0.82–1.27) and independently of the latitude (lower latitudes HR 1.05; 95% CI, 0.84–1.32 higher latitudes HR 1.16; 95% CI, 0.85–1.58). Accordingly, the study concluded that there is no evidence that higher SPF values confer greater protection against cSCC. Nevertheless, these findings may be influenced by confounding factors, such as differences in sunscreen application practices and the fact that non-users or users of lower-SPF may represent a lower-risk group (due to less sun exposure and higher phototypes) compared to users of SPF > 15 (HR 0.71; 95% CI, 0.54–0.94). Other limitations include the lack of data on the quantity and frequency of application, duration of exposure, sun avoidance behaviour and use of protective clothing.

Exposure to UV radiation is estimated to account for approximately 65% of melanoma cases, although its precise role in its pathogenesis remains incompletely understood [13]. Despite the linear relationship between the number of lifetime sunburns and melanoma development, there is ongoing debate regarding the effectiveness of ultraviolet B (UVB)-only photoprotection in melanoma prevention. This controversy arises from evidence suggesting a potential association between ultraviolet A (UVA) exposure and melanoma risk, primarily derived from animal studies [13,14]. Sunscreens that primarily block UVB radiation may paradoxically increase overall sun exposure by preventing sunburn, thereby allowing greater cumulative UVA exposure. Moreover, insufficient broad-spectrum protection during recreational sun exposure has been shown to cause more extensive UV-induced skin damage than a single indoor tanning session—even in the absence of visible sunburn—highlighting the critical importance of using broad-spectrum sunscreens [13].

Given that a high number of melanocytic nevi is a well-established risk marker for melanoma, a randomized controlled trial demonstrated that regular application of broad-spectrum sunscreen to sun-exposed areas reduced the number of nevi in freckled children by 30–40% and significantly decreased the total number of acquired nevi across all participants [15]. However, recent meta-analyses have not demonstrated a statistically significant association between sunscreen use and melanoma prevention, although the included studies were highly heterogeneous and some did not evaluate broad-spectrum formulations [13,16,17].

Regarding photoprotective behaviours among individuals newly diagnosed with skin cancer, Navarro-Bielsa et al. reported in a case–control study that sunscreen use was the most commonly adopted photoprotective measure (used “usually” or “always” by 58.9% of participants), following avoidance of sun exposure between 12:00 and 16:00 (63.1%) [18]. Melanoma patients reported less frequent use of shade and protective clothing (*p* < 0.05), whereas those with BCC or cSCC were more likely to wear hats or caps (*p* = 0.01) and to have experienced greater cumulative sun exposure over the previous 15 years. Controls reported having used sunscreens with SPF > 20 more often in the past than other cases; however, at the time of the study, all groups reported regular use of SPF > 20, and most reported SPF > 50. No significant differences were observed in photoprotection habits between participants with or without a prior history of skin cancer.

While studies primarily assess the efficacy of UV filter-based sunscreens, increasing attention has been directed toward the potential additive or complementary role of non-filtering photoprotective ingredients incorporated into some formulations. These agents are discussed separately in the following section.

### 3.2. The Role of Photoprotective Ingredients (Pings) in the Prevention of Skin Cancer

Photoprotective INGredients (PINGs) are non-filtering agents that increase the skin’s intrinsic defence against solar radiation [19]. Unlike UV filters, PINGs do not primarily act by blocking or absorbing UVRActing through DNA repair, antioxidant, immunomodulatory, anti-inflammatory, and pigmentation-regulating mechanisms; PINGs contribute to prevent or repair photodamage. They have also received the name of “biological photoprotection” with the aim to complement the UV filters, reducing the necessity to increase their concentrations.

### 3.3. DNA Repair Enzymes

This group includes photolyase, endonuclease V (T4V5) and 8-oxoguanine glycosylase (OGG1) which repair the damage to DNA caused by UVR [20].

Photolyase is a flavoprotein enzyme found in algae, fish, amphibians and some non-placental mammals, but not in humans. It recognizes damaged cyclobutane pyrimidine dimers (CPDs) and converts them into their monomeric form by directly absorbing blue light (300–500 nm), performing accurate and error-free repair, a mechanism known as photoreactivation [21]. It has been shown to be effective when applied twice daily for 6 months in 45 patients with AK, reducing the AKASI score from a mean of 2.55 to 1.90 (*p* < 0.001) and decreasing their severity, with a complete clearance of 26% of the lesions [22]. A randomized factorial clinical trial, compared a photolyase-containing sunscreen with a conventional sunscreen (both SPF 99) in the treatment of photodamage and field cancerization [23]; both treatments were applied for 8 weeks in 40 patients with AK on the forearms. In addition, they analyzed the complementary action of a topical antioxidant formula (vitamin C 15%, alpha-tocopherol 1% and ferulic acid 0.5%) versus placebo, applied at night for eight weeks. The authors concluded that the incorporation of photolyase into a high-SPF sunscreen did not provide additional benefits for field cancerization or AK, whereas topical antioxidants showed complementary usefulness in reducing lesion counts.

T4 endonuclease 5 (T4N5) is a DNA repair enzyme initially isolated from Escherichia coli infected by bacteriophage T4. It exerts its action by enhancing the nucleotide excision repair (NER) mechanism present in humans, recognizing areas of DNA damage, excising them and synthesizing a new strand with the insertion of the correct base pairs [21]. This DNA repair enzyme demonstrated ability in vitro and in vivo to counteract the harmful effect of UV light on XP patients, demonstrating after 12 months of use a 68% reduction in the incidence of AK and a 29.6% reduction in BCC in the group treated with T4N5 [24,25]. In healthy volunteers, a formulation with T4N5 endonuclease derived from *Micrococcus luteus* was used on 24 participants and one with photolyase on the other 24, with each subject acting as their own control. Retroauricular skin was exposed to UVB radiation without prior application of sunscreen, and skin samples were obtained by tape stripping before and 24 h after irradiation. Subsequently, the participants applied the repair enzyme formulations daily to the irradiated area for two weeks, after which sampling was repeated to analyze changes in gene expression. Of the 18 genes analyzed, eight showed significant changes in expression after UVB exposure; however, neither the endonuclease cream nor the photolyase cream significantly altered these patterns compared to the control. These results suggest that, although UVB radiation causes acute genetic alterations related to photoaging and skin carcinogenesis, the short-term impact of DNA repair enzymes on gene expression has not been demonstrated [26].

8-oxoguanine DNA glycosylase 1 (OGG1) is an enzyme present in bacteria, fungi, plants, and mammals that recognizes and initiates the repair of DNA damage caused by reactive oxygen species. Its main function is to identify and repair mutations in the 8-oxo-7,8-dihydroguanine (8-oxoG) base, one of the most common types of oxidative damage to DNA. In addition, it has a high affinity for mitochondrial DNA, reduces MMP-1 secretion and prevents the loss of type III collagen. Given that OGG1 expression is decreased in BCC cells [27], a preliminary study suggests that topical application of OGG1—together with photolyase, endonuclease, and antioxidants—may reduce CPD formation, protein carbonylation (PC) and 8-oxoG levels, thereby helping to prevent skin cancer [28]. To date, there are no studies evaluating the isolated topical application of OGG1. However, in 2023, a double-blind, quasi-experimental study [28] was conducted on 12 healthy volunteers to compare the efficacy of different commercially available topical formulations against skin damage induced by UV radiation. Several combinations of sunscreens were evaluated: a sunscreen with 1% photolyase (without SPF specification), a sunscreen with SPF 100 and photolyase, and another with SPF 100 that also included photolyase, endonuclease, and OGG1. Additional comparators included piroxicam, formulations with urea, lactic acid and octatrienoic acid, and a placebo. The results showed that the sunscreen with 1% photolyase was the most effective in reducing CPDs, followed by the sunscreen with SPF 100 plus photolyase and the one that also contained endonuclease and OGG1. In terms of PC reduction, the formulation with SPF 100, photolyase, endonuclease, and OGG1—which also included antioxidants—was the most effective. However, this study did not evaluate the impact of these formulations on AK.

Recently, our group has published a systematic review [21] on the topical use of repair enzymes for the prevention and treatment of precancerous lesions and skin cancer. The review included 20 studies: 8 with photolyase, 6 with endonuclease, and 6 with combinations of different DNA repair enzymes. Photolyase reduced CPDs by 40–93% in the studies that analyzed them and demonstrated clinical, dermatoscopic and histological improvement in AK in most studies, except for one study that showed no significant changes. In contrast, endonuclease did not produce a statistically significant reduction in CPDs compared with placebo, although a decrease in AK was observed in three of four studies. Combinations of DNA repair enzymes reduced CPDs by 47–67% in all studies. The addition of antioxidants together with repair enzymes increased efficacy. It should also be noted that most studies were conducted in patients already diagnosed with AK or KC, whereas six studies were performed in the general population.

Furthermore, the development of nanoencapsulated systems, smart formulations, and personalized approaches based on skin phototype or OGG1 polymorphisms are expected to be the next DNA repair lines of advancement in active photoprotection [20].

### 3.4. Antioxidants

Exposure to solar radiation, mainly UV, generates oxidative stress in the skin through the formation of free radicals and reactive oxygen species (ROS), contributing to DNA damage, photoaging, immunosuppression, and skin carcinogenesis. Antioxidants, both endogenous and exogenous, have emerged as key tools for preventing and mitigating these effects, acting as modulators of oxidative pathways and enhancers of DNA repair systems [29,30].

*Polypodium leucotomos* extract (PLE, Fernblock^®^) is the antioxidant with the most evidence of its preventive effect on skin cancer. Its antioxidant activity is mainly due to its high polyphenol content, which neutralizes ROS, reduces lipid peroxides and regulates redox signalling pathways such as NRF2 and MAPK [30]. PLE has been shown to protect against oxidative DNA damage, preventing the formation of CPDs during and after UV exposure (“dark-CPDs”), as well as reducing 8-OH-dG and other markers of oxidative stress [31]. Furthermore, PLE has been shown to modulate the expression of TNF-α, COX-2 and NF-kB, reducing UV-induced inflammation and melanin production [29,30].

In a randomized, multicentre clinical trial [32], 131 patients with severe actinic damage were divided into three groups: one applied daily sunscreen SPF 50+, the other applied the same sunscreen but with PLE added, and the third one applied the same sunscreen with PLE and also took oral PLE 240 mg/day. The control group treated with sunscreen alone showed a 3% increase in the AKASI at 6 months, with no further change at 12 months, whereas the group treated with both topical and oral PLE showed a 7% decrease at 12 months (*p* = 0.001). Furthermore, the AK-FAS index, indicative of hyperkeratinisation, increased from 9.3% to 30% at 12 months in the control group, whereas it decreased from 13.6% to 5.9% in the group treated with the topical PLE and from 13.6% to 3% in the group treated with both topical and oral PLE at 12 months. In addition, the appearance of new lesions at 6 months was limited to 2.6% in the group treated with topical PLE and was non-existent in the group treated with both topical and oral PLE, compared to 25% of the controls who developed at least one new actinic keratosis (*p* = 0.008).

In another study, the oral administration of PLE (960 mg per day for 1 month and then 480 mg per day for 5 months) one week after photodynamic therapy with methyl-aminolevulinate (MAL-PDT) statistically significantly reduced development of scalp AK compared to the control group [33].

Particularly interesting is a study conducted on 18 patients with XP who, apart from receiving a photoeducation programme, were supplemented with 960 mg of PLE daily and vitamin D along with the application of SPF 50+ every two hours of sun exposure. Eleven did not develop any lesions after one year of treatment (61%). The remaining seven patients developed 12 new lesions, of which 5 were AK, 4 were BCC, and 1 was a SCC in situ. It is noteworthy that 60% of patients who did not develop lesions exhibited ideal photoprotection behaviour, whereas only 30% of those who developed lesions adhered properly to photoprotection guidelines [34].

Finally, PLE has demonstrated in vitro protective effects against visible light, infrared radiation and environmental pollution. These exposures are known to induce oxidative stress, increase MMP-1 expression, and promote melanogenesis and proinflammatory cytokine production, all of which may enhance photocarcinogenic pathways. PLE has been shown to attenuate MMP-1 overexpression induced by visible and infrared radiation, modulate melanin synthesis and inflammatory responses, and reduce pollutant-associated oxidative damage. By counteracting these molecular alterations, PLE may help mitigate the synergistic carcinogenic effects resulting from the interaction between UVR and environmental pollution [30,35].

Vitamin C (ascorbic acid) is a water-soluble antioxidant that neutralizes free radicals generated by UV, visible and infrared radiation and contributes to the regeneration of vitamin E [36].

Regarding the use of vitamin C as a treatment for non-melanoma skin cancers, we found two studies in patients with BCC and another with SCC. Holló et al. [37] treated 7 BCCs in 6 patients by applying 33% vitamin C under occlusion for 12 h daily over a period of 22 weeks. Most of the tumours were superficial (n = 6), and one was nodular (n = 1). The nodular BCC showed a complete clinical and histological response, whereas among the superficial tumours, four achieved a complete response and two achieved a partial response. With a mean follow-up of 18 months, no recurrences of nodular BCC were observed and only one superficial case relapsed. Adverse effects were limited to mild skin irritation. Subsequently, a randomized clinical trial treated 15 BCCs (11 nodular and 4 superficial) in 13 patients with 30% vitamin C in dimethyl sulfoxide (DMSO) applied twice daily, comparing them with 14 BCCs treated with 5% imiquimod for 8 weeks. In the vitamin C group, 86.7% of cases showed complete clinical and histological resolution, and 13.3% showed partial resolution, with no recurrence after an average follow-up of 2.5 years, compared to 57.1% with imiquimod (*p* < 0.05) [38]. The side effects of vitamin C in DMSO were mainly pruritus (57%), mild pain (8%) and skin erosion (16%), with no residual hypopigmentation, compared to the group treated with imiquimod, where hypopigmentation was observed in 70% of cases. Additionally, one well-differentiated invasive SCC located on the antihelix was treated with a supersaturated solution of vitamin C (40–70%) under occlusion for 4 to 12 h daily for 30 days [39]. After treatment, the tumour was excised observing clinical and histological complete resolution with no recurrence or adverse effects.

Vitamin E (tocopherol) is a fat-soluble antioxidant found in eight isoprenoid residues that protects cell membranes from lipid peroxidation caused by ROS induced by solar radiation. Its combination with vitamin C enhances the photoprotective effects, as vitamin C regenerates the active form of vitamin E after the neutralization of free radicals [36]. The most common form found in food is gamma-tocopherol; however, alpha-tocopherol is the most common form in the body and is considered the main isomer. In vitro and mice studies during the 2000s showed anticarcinogenic effects [40,41]. A case–control study suggested that vitamin E intake might have a protective role in the development of BCCs [42]; subsequent evidence from a double-blind, placebo-controlled study indicates that oral administration of 400 IU of alpha-tocopherol acetate over a 6-month period neither produced histological differences nor affected the minimal erythema dose in response to UVB [43]. These findings suggest that, despite modest increases in plasma vitamin E levels, oral supplementation does not confer meaningful photoprotection.

Green tea and black tea extracts, rich in catechins and flavonoids, have also been studied for their antioxidant and anti-inflammatory properties. There are studies showing that oral or topical administration of tea polyphenols decreases the formation of UV-induced CPDs in keratinocytes and fibroblasts, reduces inflammation by inhibiting NF-kB and COX-2, and improves DNA repair by modulating nucleotide repair enzymes [44]. However, a randomized placebo-controlled clinical trial conducted with 50 participants supplemented orally with green tea sinecatequins for 3 consecutive months did not show significant differences in skin erythema, leukocyte infiltration, or eicosanoid response after stimulation with UV radiation [45]. Nevertheless, the Singapore Chinese Health Study [46], where 63,257 participants were followed for 5 years, found that black tea drinkers had a decreased risk of KC compared to non-drinkers (HR, 0.70; 95% CI, 0.52–0.94). They did not find a statistically significant relationship with green tea intake.

Caffeine, used both orally and topically, has shown significant effects in photoprotection and skin cancer prevention. Preclinical studies show that topical application of caffeine increases apoptosis of UVB-damaged keratinocytes and promotes the elimination of precancerous cells. In murine models, caffeine reduced UVB-induced wrinkle formation by more than 35% and increased the sun protection factor (SPF) by 25% when combined with sunscreens [47]. A meta-analysis conducted on coffee consumption in the US population found that any coffee consumption compared to non-coffee drinkers was protective against the onset of melanoma (RR = 0.89; 95% CI: 0.80, 0.99) and against the onset of KC (RR = 0.92; 95% CI: 0.89, 0.95) [48]. Another meta-analysis showed a reduction in the risk of melanoma among consumers of caffeinated coffee (RR = 0.82, 95% CI: 0.69–0.97), whereas no significant differences were found when it came to decaffeinated coffee consumption (RR = 0.94, 95% CI: 0.82–1.08) [49]. In the Mendelian randomization study by Liu et al. [50] that evaluated the causal relationship between different risk factors and cutaneous melanoma in 30,134 patients, no significant association was found between genetic predisposition to coffee consumption and the risk of developing the disease (IVW OR = 0.827; 95% CI 0.609–1.124, *p* = 0.225). This approach uses genetic variants associated with coffee consumption behaviour as instruments to infer causality, reducing the biases inherent in observational studies. Finally, two recent studies from our group that analyze all the possible factors of the exposome of BCC and SCC, using the detailed dietary questionnaire PREDIMED, found that the intake of 2–3 cups of caffeinated coffee every day was the only dietary protective factor found in both tumours [51,52].

Omega-3 fatty acids (EPA/DHA) modulate UV-induced inflammation and skin immunosuppression. In a randomized clinical trial, 79 adults supplemented with 5 g/day of EPA for one month showed less immunosuppression and lower PGE_2_ production after controlled UV exposure [53]. In organ transplant recipients patients, the intake of high doses of total long-chain omega-3 PUFA was associated with a significantly lower risk of SCC (RRadj 0.33, 95% CI 0.18–0.60), and those taking higher amounts of a-linolenic acid showed a significantly lower incidence of BCC (RRadj 0.40, 95% CI 0.22–0.74) [54]. However, in our studies of the exposome of BCC and SCC, the intake of linolenic acid was the only dietary factor associated with an increased risk of both tumours in non-immunocompromised patients [51,52].

Nicotinamide, also known as niacinamide or vitamin B3, is not only a molecule with antioxidant power but also promotes DNA repair and has anti-inflammatory properties [55]. UV radiation promotes overactivation of poly-ADP-ribose-polymerase-1 (PARP-1), resulting in intracellular NAD^+^ and ATP depletion and subsequent impairment of DNA repair mechanisms. By restoring NAD^+^ availability and preserving cellular energy balance, nicotinamide enhances nucleotide excision repair and facilitates the clearance of UV-induced DNA photolesions, including cyclobutane pyrimidine dimers [56]. In human keratinocytes isolated from biopsies of patients with precancerous lesions, nicotinamide reduced the levels of ROS and the expression of the enzyme oxoguanine glycosylase 1, protecting against oxidative damage induced by UVB radiation [57].

Topical application of broad-spectrum sunscreen containing 2% nicotinamide and panthenol (SSNP) on photoaged skin of the forearms in 14 subjects for 4 weeks found 5429 differentially expressed genes were identified after treatment. In terms of signalling pathways, significant inhibition of multiple proinflammatory and oxidative stress response pathways involved in the skin’s response to UV radiation damage was observed, although the differences were not statistically significant. Finally, a randomized clinical trial including 26 patients with 95 KA, topical 5% fluorouracil daily for 4 weeks was compared with topical 1% niacinamide twice daily for 3 months. In the group treated with topical fluorouracil, 37.2% of lesions showed mild to moderate improvement and 62.8% showed good to excellent improvement, compared with 68.1% and 31%, respectively, in the group treated with niacinamide. However, adverse effects were more frequent in the fluorouracil-treated group than in the nicotinamide group, where they were rare [58].

Resveratrol, an antioxidant found in grapes and other plants, has been shown to protect the skin from UV radiation by activating sirtuins and improving DNA repair, reducing ROS and regulating NF-kB and inhibiting MMPs, and preserving the extracellular matrix against photoaging [29]. However, there are still no clinical studies to support its efficacy as a preventive agent for AK and KC.

### 3.5. Oral Photoprotection

A recent systematic review analyzed 21 studies published between 2013 and 2023 on the role of oral supplements in preventing and treating AK as well as areas of field cancerization [59]. The studies were classified into three groups: polyphenols (11 studies), vitamins (8 studies), and other supplements (2 studies), including 12 clinical trials and 9 preclinical trials. This review found that polyphenols, especially PLE and combinations of tea, rosemary and citrus polyphenols, showed both preventive and therapeutic efficacy. Preclinical studies showed that PLE and other polyphenols decrease the formation of CPDs, regulate p53/p21 expression, reduce oxidative stress and improve the regenerative capacity of keratinocytes, helping to protect against UV-induced damage and skin aging.

Regarding vitamins, nicotinamide showed consistent benefits in reducing new AK and KC whereas other vitamin supplements showed inconsistent results. A retrospective study of more than 33,000 patients in the US Veterans Health Care System showed a 14% reduction in the risk of developing new skin cancers in those treated with nicotinamide (500 mg twice daily for more than 30 days) compared to those who were not treated. This effect was more pronounced when treatment was started after the first skin cancer, reaching a 54% reduction. However, the benefits decreased with each additional diagnosis of skin cancer [60].

A systematic review identified four randomized double-blind clinical trials that evaluated oral nicotinamide doses of 500 mg twice daily for 4 to 12 months in high-risk patients with a history of at least two KC. The results showed that, overall, nicotinamide consumption was not significantly associated with a reduction in the risk of SCC (RR 0.81, 95% CI 0.48–1.37), BCC (RR 0.88, 95% CI 0.50–1.55), or KC overall (RR 0.82, 95% CI 0.61–1.12), especially when combining immunocompetent and immunosuppressed patients. However, nicotinamide was found to have a protective effect on the incidence of AK and KC when only the immunocompetent patient group was considered [61]. This lack of beneficial effect of oral nicotinamide on organ transplant recipients was confirmed in the ONTRANS randomized clinical trial [62]. The lack of efficacy appears to be explained by the interference of classic immunosuppressants (tacrolimus, azathioprine, mycophenolate, cyclosporine), which block DNA repair enzymes and neutralize the photoprotective mechanism of nicotinamide. Other limitations were slow recruitment and a lower-than-expected tumour incidence (2.6 SCs/patient/year). Even so, nicotinamide showed an excellent safety profile and good long-term tolerance, with no metabolic, renal alterations or even major adverse cardiovascular events [63].

A meta-analysis compiled results from various studies conducted with oral nicotinamide supplementation in severely sun-damaged patients, whether immunocompetent or immunocompromised [61]. They indicate that the preventive impact of nicotinamide on KC and AK is not maintained in the long term, with normalization of the compared groups after 6 months of stopping the drug.

These findings highlight the complexity of nicotinamide’s role in skin cancer prevention and underscore the need for further research to understand its mechanisms of action, its actual efficacy, and to assess the possible adverse effects of long-term use.

Therefore, although it cannot be recommended as effective photoprotection in organ transplant recipients, nicotinamide remains a safe and theoretically plausible adjuvant in patients with high sun exposure or under less genotoxic immunosuppressive regimens, such as mTOR inhibitors. Its use could be re-evaluated in combination strategies alongside intensive topical photoprotection and close dermatological surveillance [62].

Vitamin D is an essential fat-soluble micronutrient that comes in two main forms, D2 (ergocalciferol) and D3 (cholecalciferol), which are only obtained in small amounts from diet. It is known that pre-vitamin D is converted into D3 in the skin thanks to UVB radiation, then undergoes two hydroxylations, renal and hepatic, until it reaches its active form 1,25(OH)2D (calcitriol). Calcitriol has been shown to protect against photodamage by repairing CPDs, mitigating oxidative stress and reducing chronic inflammation [64].

In a randomized, placebo-controlled clinical trial conducted in elderly Australians, participants were supplemented monthly with 60,000 IU of cholecalciferol for 5 years [65]. The researchers found that vitamin D supplementation did not reduce the number of KC removed or AK treated, nor did it delay the time to onset of the first KC. In fact, exploratory analysis in individuals aged ≥ 70 years showed a trend towards an increase in SCC. Therefore, vitamin D should not be recommended for the chemoprevention of SCC in the general elderly population.

An overview of topical and oral photoprotective ingredients, their mechanisms of action, and available clinical evidence is provided in Table 1.

### 3.6. The Effect of Visible Light in the Skin

Human skin pigmentation is a complex and strictly regulated biological process, primarily stimulated by solar radiation. Although most of the known effects are attributed to UV radiation, it accounts for only 2–5% of the solar spectrum. In contrast, the effects of visible light, which represents nearly half of the solar spectrum, remain largely unexplored. Visible light has been shown to activate matrix metalloproteinases and reduce collagen synthesis through oxidative stress mechanisms, also inducing more intense and longer-lasting pigmentation than UVA1 [66].

Although short-term exposure to blue light does not cause any significant deleterious effects on the skin, and despite its per-photon efficacy in glutathione oxidation being only 25% that of UVA, blue light is twice as abundant in the solar spectrum as UVA. Consequently, its overall contribution to ROS production can be estimated to be approximately half that of UVA [67]. Conversely, blue light at 453 nm, when applied to previously damaged skin, has been shown to reduce inflammation (↓ IL-1α) but delay barrier recovery (↑TEWL) and induce mild pigmentation within 72 h [68].

In addition, blue light exhibits a synergistic interaction with UVA1, particularly regarding the induction of hyperpigmentation, suggesting that the UVA–visible light boundary (380–420 nm) may participate in photoinduced reactions extending beyond the classical UV range [69]. This region has also been linked to delayed DNA damage, oxidative stress and gene expression changes related to photoaging and inflammation. Recent studies show that formulations including filters targeting this spectral transition significantly reduce photodamage, highlighting the need for extended broad-spectrum coverage [70].

Regarding carcinogenesis, studies using a reconstructed human epidermis model have shown that blue light (427 ± 30 nm, 60–92 J/cm^2^) does not directly induce CPDs or 6-4 photoproducts (64PPs), but it significantly reduces—by 48%—the repair efficiency of UVB-induced DNA damage, particularly of CPDs. This finding suggests an indirect genotoxic interference of blue light with the NER pathway, possibly mediated by redox alterations. Furthermore, photoprotection during exposure using a formulation containing or an SPF 50+ sunscreen with UVB, UVA and blue light filters (TriAsorB^®^ 5%) doubled the repair efficiency of TT-CPDs and improved that of TC-CPDs and 64PPs, supporting the preventive convenience of filtering high-energy visible (HEV) light under conditions of intense solar exposure [71].

Chronic exposure to blue light (408 nm, 50 J/cm^2^—equivalent to approximately 60 min of sunlight per session, three times per week) in human keratinocytes (HaCaT) over a total of 42 irradiations induced progressive morphological and molecular alterations: enlarged and irregular nuclei, chromatin aggregation, multinucleation, increased proliferation (bromodeoxyuridine incorporation), and resistance to apoptosis. At the transcriptional level, acute exposure (three irradiations) upregulated genes related to keratinization and downregulated those involved in tissue repair and apoptosis, modulated by transcription factors such as IRF1, EGR1, ELF3, and FOSL1, which are typically associated with the UVB photostress response. In contrast, chronic exposure (42 irradiations) promoted the activation of metabolic and oxidative phosphorylation (OXPHOS) pathways, together with the suppression of immune and inflammatory responses, regulated by CENPX, SRF, CEBPB, and KLF4. Although complete malignant transformation was not achieved, these findings support that high-energy visible (HEV) light may act as a tumourigenic cofactor in the presence of preexisting genetic damage, such as the p53 mutation [72].

In hairless mice chronically exposed for one year to visible light of different wavelengths (blue 479 nm, green 538 nm, and red 629 nm), small cutaneous tumours developed exclusively under blue light exposure (40 kJ/m^2^ per day; equivalent to 30–60 min of sunlight). This response was associated with increased epidermal proliferation markers (Ki-67, cyclin D1) and a neutrophil-driven inflammatory microenvironment characterized by NETosis (CXCR1, citH3, PAD4, elastase, neutrophilic ROS), M1 macrophage infiltration, and elevated systemic IL-6 and IL-23. Collectively, these findings point to a spectrally specific, inflammation–oxidation-mediated pathway with potential tumourigenic relevance [73].

However, there is no evidence in humans that visible light induces or participates in the cutaneous carcinogenic process.

**Table 1:** Clinical and experimental evidence on photoprotective ingredients, mechanisms of action, study designs, and reported outcomes.

PING	Mechanism	Study Design	Results	Author
Photolyase	CPDs repair by absorbing blue light (photoreactivatión)	Cohort (n = 45) Photolyase-containing sunscreen BID for 6 months	↓ AKASI AK complete clearance: 26%	Bulla et al. 2025 [22]
		RCT (n = 40) Photolyase sunscreen vs. regular sunscreen BID for 8 weeks	No additional benefits	Alvares et al. 2022 [23]
T4N5	Enhancing NER mechanism	RCT (n = 30, XP) T4N5 liposome lotion vs. placebo lotion Daily for 1 year	↓ 68% AK incidence ↓ 30% BCC incidence	Yarosh et al., 2001 [24]
		RCT (n = 48) T4N5 BID for 2 weeks vs. photolyase cream daily for 2 weeks vs. control	No impact on gene pattern expression	Anderson et al., 2023 [26]
OGG1	Repair 8-oxoG base mutations caused by ROS, ↓ MMP-1, ↓ type III collagen loss.	Experimental (n = 12) Mixtures of PING (DNA repair enzymes, antioxidants, keratolytic agent…)	↓ CPD effectiveness: 1% liposomal photolyase > encapsulated photolyase SPF 100 > photolyase, endonuclease, glycosylase OGG1 SPF 100	Minoretti et al., 2023 [28]
PLE and other polyphenols	Neutralize ROS ↓ CPDs, MMP-1 and 8-OH-dG ↓ UV, VIS and IR-induced inflammation and melanin production	RCT (n = 131, SAD) BID topical PLE + sunscreen vs. daily oral PLE + sunscreen vs. nonspecific sunscreen for 1 year	↓ AKASI, hyperkeratinisation and appearance of new lesions, especially in oral PLE group	Pellacani et al., 2023 [32]
		NRCT (n = 34, AK + PDT) Daily oral PLE + sunscreen vs. sunscreen for 5 months	↓ AKASI incidence	Auriemma et al., 2015 [33]
		Cohort (n = 18, XP) Daily oral PLE + topical PLE sunscreen for 1 year	60% did not developed any lesions at 12 months 70% of those who developed lesions had an ideal photoprotective behaviour	El Mansouri et al., 2023 [34]
		SR (9 preclinical and 12 clinical trials) Polyphenolic compounds intake (including PLE)	Clinical and preclinical evidence of reducing AK Protective effects are compound-specific (not universal across all polyphenol sources)	Rodriguez-Luna et al., 2025 [59]
VITAMIN C (ascorbic acid)	Neutralize UV, VIS and IR-induced ROS Contributes to tocopherol regeneration	Case series (n = 7, BCC) Daily 33% topical vitamin C for 22 weeks	5 complete responses 2 partial responses 1 relapse	Holló et al., 2016 [37]
		RCT (n = 25, BCC) 30% topical vitamin C BID vs. 5% imiquimod 5 days a week for 8 weeks	86,7% complete response (superior to topical imiquimod) Less adverse effect (no hypopigmentation)	Burke et al., 2022 [38]
		Case report (SCC) 40–70% Vitamin C daily for 30 days	Complete resolution of the tumour	Pernice et al., 2020 [39]
VITAMIN E (tocopherol)	Protects cell membranes from lipid peroxidation caused by UV-induced ROS	Case–control 1:2 (n = 370, NMSC) 3.06 mg vitamin E daily	Protective effect OR of BCC = 0.731	Davies et al., 2002 [42]
		RCT (n = 12) 400 IU of oral vitamin E daily vs. placebo	No differences in the response to UVB	Werninghaus et al., 1994 [43]
TEA EXTRACTS	↓ UV-induced CPDs and inflammation (NF-Kb and COX-2) ↑ DNA repair (NER)	RCT (n = 50) Oral green tea catechins vs. placebo BID for 3 months	No differences in the response to UV radiation.	Farrar et al., 2015 [45]
		Cohort (n = 63,257)	Black tea drinkers: ↓ KC (HR = 0.70) Green tea drinkers: no significant relationship	Oh et al., 2019 [46]
CAFFEINE	↑ Apoptosis of UVB-damaged keratinocytes	MA of cohorts (n = 3,713,932) Caffeinated coffee consumption	↓ RR of melanoma (0.89) and NMSC (0.92)	Di Maso et al., 2021 [48]
		MA of cohorts (n = 1,418,779) Coffee consumption	↓ RR of melanoma (0.82)	Micek et al., 2017 [49]
		Mendelian randomization (n = 30,134) Genetic predisposition to coffee consumption	No significative association with risk of melanoma	Liu et al., 2023 [50]
		Case–control 1:2 (n = 188, SCC)	Caffeinated coffee intake was higher in controls than in SCC patients (3.55 vs. 2.5 coffees per day, *p* = 0.01)	Navarro-Bielsa et al., 2023 [52]
		Case–control 1:1 (n = 246, BCC)	Caffeinated coffee intake was higher the controls than in BCC patients (3.55 vs. 2.88 coffees/day; *p* = 0.05)	Navarro-Bielsa et al., 2024 [51]
OMEGA-3 (EPA/DHA)	↓ UV-induced inflammation and immunosuppression	RCT (n = 79) 5 g n-3 PUFA containing lipid (70% EPA + 10% DHA) vs. control lipid daily for 3 months	↓ immunosuppression and PGE2 production after UV exposure	Pilkington et al., 2013 [53]
		Cohort (n = 449, SOTR)	High doses of total long-chain omega-3 PUFA: ↓ risk of SCC (RR = 0.33) High doses of alpha-linolenic acid: ↓ risk of BCC (RR = 0.40)	Miura et al., 2020 [54]
		Case–control 1:2 (n = 188, SCC)	Patients with SCC had a higher linolenic acid intake vs. controls (1.89 vs. 1.40, mcg/day *p* = 0.04)	Navarro-Bielsa et al., 2023 [52]
		Case–control 1:1 (n = 246, BCC)	Patients with BCC had a higher linolenic acid intake than controls (1.74 vs. 1.40 μg/day; *p* = 0.02)	Navarro-Bielsa et al., 2024 [51]
NICOTINAMIDE	↓ ROS y oxoguanine glycosylase 1 expression	Experimental (n = 14, SAD) SSNP daily for 4 weeks, pre vs. post treatment vs. no SAD	↑ collagen synthesis ↓ cGMP-PKG and MAPK signalling pathways activated by UV exposure	Torres-Moral et al., 2024 [74]
		Cohort (n = 33,822) Nicotinamide 500 mg BID for more than 30 days	↓ risk of new skin cancer: 14%	Breglio et al., 2025 [60]
		MA of 4 RCT (KC) Nicotinamide 500 mg BID for 4–12 months	No significant association with ↓ risk of SCC, BCC nor KC overall. ↓ new AK and KC in immunocompetent patient group alone	Tosti et al., 2023 [61]
		RCT (n = 26, AK) topical 1% niacinamide BID for 3 months	Mild-moderate improvement: 68.1%, good to excellent improvement: 31%	Poostiyan et al., 2025 [58]
VITAMIN D (cholecalciferol)	Repairing CPD, ↓ oxidative stress and chronic inflammation	RCT (n = 20,334) 60,000 UI cholecalciferol monthly for 5 years	No ↓ KC nor AK A trend towards ↑ SCC	Ali et al., 2022 [65]

Abbreviations: AK, actinic keratosis; AKASI, Actinic Keratosis Area and Severity Index; BCC, basal cell carcinoma; BID, twice daily; CI, confidence interval; COX-2, cyclooxygenase-2; CPD, cyclobutane pyrimidine dimer; EPA, eicosapentaenoic acid; HR, hazard ratio; IR, infrared radiation; KC, keratinocyte cancer; MAL-PDT, methyl aminolevulinate photodynamic therapy; MAPK, mitogen-activated protein kinase; MED, minimal erythema dose; MMP-1, matrix metalloproteinase-1; MR, Mendelian randomization; NER, nucleotide excision repair; NF-κB, nuclear factor kappa B; NRCT, non-randomized clinical trial; NMSC, non-melanoma skin cancer; OGG1, 8-oxoguanine DNA glycosylase 1; OR, odds ratio; PDT, photodynamic therapy; PINGs, photoprotective ingredients; PLE, *Polypodium leucotomos* extract; PUFA, polyunsaturated fatty acids; RCT, randomized controlled trial; ROS, reactive oxygen species; RR, relative risk; SAD, severe actinic damage; SCC, squamous cell carcinoma; SPF, sun protection factor; SOTR, solid organ transplant recipients; SSNP, sunscreen containing nicotinamide and panthenol; TNF-α, tumour necrosis factor alpha; UVA, ultraviolet A radiation; UVB, ultraviolet B radiation; UVR, ultraviolet radiation; VIS, visible light; XP, xeroderma pigmentosum. ↓ (DECREASE) and ↑ (INCREASE).

## 4. Conclusions

Photoprotection today must be understood as a multidimensional strategy that integrates UV filtering, visible light protection, DNA-repair mechanisms, antioxidant support, and behavioural interventions tailored to individual risk. Keeping in mind that photoprotection behaviour includes avoiding sun exposure during the midday, using shades, clothing hats and sunglasses, evidence demonstrates that daily sunscreen use, particularly broad-spectrum formulations, reduces AK and SCC and may contribute to melanoma prevention, especially in populations at risk. Biological photoprotectors—including DNA-repair enzymes and antioxidants—provide meaningful complementary benefits by mitigating oxidative stress, enhancing DNA repair, and improving subclinical actinic damage. Nicotinamide remains a promising adjuvant in immunocompetent individuals with high cumulative sun exposure. The growing recognition of the harmful effects of visible light, especially HEV light, in skin reinforces the need for expanded spectral protection and for further investigation. In high-risk groups such as transplant recipients, XP patients, people with albinism, and outdoor workers, structured education and adherence–reinforcement programmes significantly improve outcomes.

## 5. Future Directions

Despite major advances in photoprotection, important gaps remain in understanding how to optimally prevent skin cancer across populations and exposure settings. Future research should move beyond a purely UV-centric model toward a more comprehensive, mechanistic, and personalized approach.

Long-term prospective studies are needed to clarify the impact of daily photoprotection on basal cell carcinoma and melanoma, using standardized broad-spectrum formulations, objective adherence measures, and extended follow-up. In parallel, the contribution of non-UV wavelengths—particularly HEV light—requires validation in humans through epidemiological and clinical studies assessing chronic exposure and the efficacy of HEV-filtering sunscreens.

Biological and oral photoprotection strategies show promise but require stronger clinical validation. Randomized trials should better define the value of adding photoprotective ingredients beyond UV filters to sunscreens, exploring innovative delivery systems and looking for natural products respectful of human and environmental health.

Finally, adopting an exposome-based framework that integrates solar radiation with environmental, occupational, and individual susceptibility factors may enable earlier risk stratification and personalized prevention strategies.

## 6. Key Points

- Daily sunscreen use effectively reduces AK and SCC, with emerging but less conclusive evidence for BCC and melanoma prevention.
- Broad-spectrum protection (UVB, UVA, and visible light) is essential to prevent skin cancer.
- DNA-repair enzymes, especially photolyase, reduce CPDs and improve AK, whereas combinations including OGG1 may enhance protection.
- Antioxidants, notably *Polypodium leucotomos*, show consistent benefits in reducing oxidative damage, AK progression and carcinogenic risk.
- Visible light contributes to pigmentation, ROS generation, and may potentiate carcinogenesis, underscoring the need for broad spectrum sunscreens.
- Oral photoprotection is a useful adjunct, particularly polyphenols, such as PL, and selected vitamins, like nicotinamide, to prevent mostly AK but should not replace topical measures.
- Educational interventions and photoprotection campaigns are necessary to guarantee healthy behaviour of the population outdoors.

## Data Availability

No new data were created or analyzed in this study. Data sharing is not applicable to this article.
